# The temperament and character personality profile of the glaucoma patient

**DOI:** 10.1186/s12886-015-0117-9

**Published:** 2015-10-01

**Authors:** Harun Çakmak, Vesile Altinyazar, Suzan Güven Yilmaz, İmran Kurt Ömürlü, Tolga Kocatürk, Alper Yazici, Cumali Değirmenci, Sema Oruç Dündar, Halil Ates

**Affiliations:** Department of Ophthalmology, Adnan Menderes University Medical Faculty, Merkez Kampus Kepez Mevkii, 09100 Aytepe, Aydın Turkey; Department of Psychiatry, Adnan Menderes University Medical Faculty, Merkez Kampus Kepez Mevkii, 09100 Aytepe, Aydın Turkey; Department of Statistic, Adnan Menderes University Medical Faculty, Merkez Kampus Kepez Mevkii, 09100 Aytepe, Aydın Turkey; Department of Ophthalmology, Ege University Medical Faculty, Ege Universitesi Tip Fakultesi Hastanesi, 35040 Bornova, Izmir Turkey; Department of Ophthalmology, Balıkesir University Medical Faculty, Usak yolu uzeri Cagis yerleskesi, 10145 Balikesir, Turkey

**Keywords:** Glaucoma, Temperament and Character inventory, Personality profile

## Abstract

**Background:**

To determine the temperament and character profile of glaucoma patients.

**Methods:**

A total of 234 patients (104 with primary open angle glaucoma, and 130 control subjects without any ocular disease) were selected for this prospective, cross-sectional study. All the participants underwent a comprehensive ophthalmological examination, including the best corrected visual acuity, intraocular pressure measurement, gonioscopy, and visual field analysis. All the participants were given the Turkish version of the Temperament and Character Inventory (TCI). The TCI is a self-reported evaluate, with 240 true/false items measuring four domains of temperament; harm avoidance (HA), persistence (PS), novelty seeking (NS), reward dependence (RD), and three domains of character; self-transcendence (ST), cooperativeness (C), self-directedness (SD).

**Results:**

The glaucoma patients achieved the higher scores than the controls for the HA and SD dimensions (*p* < 0.001 and *p* = 0.033). The glaucoma patients scored lower than the controls for the NS, P and ST dimensions (*p* < 0.001, *p* < 0.001 and *p* = 0.002). There were no differences in the RD and C scores between the patients and the controls (*p* = 0.944 and *p* = 0.343). There was no correlation between the duration of illness and the TCI dimensions. Disease severity was positively associated with HA (*r* = 0,220, *p* = 0,025) and the anticipatory worry (*r* = 0.227, *p* = 0.021) dimension.

**Conclusions:**

Glaucoma patients had a different personality profile to healthy individuals. This may affect treatment compliance and is also important when coping with maladaptive patient attitudes.

## Background

Glaucoma is characterized by progressive optic nerve damage, which is one of the most frequent cause of irreversible visual damage and blindness in the industrialized world [1]. Stress is considered as a risk factor for glaucoma, and is reported as increasing the intraocular pressure (IOP) both in healthy subjects and glaucoma patients [2, 3]. In previous studies, the importance of personality structure has proven the perception that there is a maintenance of high levels of stress in glaucoma patients [4, 5]. The differences in personality structure of glaucoma patients; excitable temperament, perfectionistic pattern, neuroticism, hypochondriacal tendencies, irritability, anxiety traits, and type A behavioral pattern introversion are the issues which have been reported on for a long time [6–13]. In some studies, these personality patterns were reported to affect adherence to treatment, and some have been reported to be a risk factor in the development of glaucoma [9, 13]. However, personality was not measured by the validity criteria, and a consistent personality profile of glaucoma patients has yet to be established.

According to Cloninger’s psychobiological model, the dimensional approach has some advantages when studying personality [14]. Personality has been proposed as two separate elements i.e. temperament and character. Temperament is proposed as the underlying biological and genetic structural component of the personality, and refers to the automatic emotional response to incidents; while character involves self-conception, and is affected by life experiences, which provide diversity in the values of individual choice [15]. According to this model, temperament is proposed as being dividing into four different independent dimensions; Novelty Seeking (NS) is the orientation towards innovation and reward. Harm Avoidance (HA) is the tendency to inhibit behavior in response to unrewarded situations, and to avoid potential punishment. Reward Dependence (RD) is the tendency to maintain behavior to be socially rewarded. Persistence (PS) is the continuation of the certain behavior in spite of the intermittent reward and frustration. Character is divided into three different dimensions; Self-directedness (SD) is the perception of the self as an autonomous individual. Cooperativeness (C) is the perception of the self as a part of society, and positive relationships with others. Self-transcendence (ST) is as part of the self-perception of the universe.

To our knowledge the Temperament and Character Inventory-240 (TCI-240), with a personality test that has shown a good genetic link, has not been used with glaucoma patients. The aim of this study was to investigate whether there was a different personality structure in glaucoma patients when compared to healthy subjects. For if such different personality structure was found to be associated with the parameters of the disease, then the treatment was to address that influence. Thus, it might be possible for a psychological intervention to be used for improving the treatment of glaucoma.

## Methods

A total of 234 patients (104 with primary open angle glaucoma [POAG], and 130 control subjects without any ocular disease) were enrolled on this prospective, cross-sectional study. All the participants underwent a comprehensive ophthalmological examination, including best corrected visual acuity, the IOP measurement with a Goldman applanation tonometer, a gonioscopy, visual fields using with the Humphrey D (Humphrey Systems Field Analyzer Model II 750, Zeiss, USA) the 24–2 Swedish Interactive Thresholding Algorithm I (Standard 24–2 VF tests; SITA-SAP, Carl Zeiss Meditec, Inc.) standard program, and a detailed fundus examination. The patients with unreliable visual fields or additional ocular abnormalities were excluded from the study.

The ethics approval for this prospective study was obtained from the Adnan Menderes University local research ethics committee. Written informed consent for participation in the study was obtained from each patient.

The participants with IOP <21 mmHg, normal visual fields (VF), normal optic discs, open angles on gonioscopy and no suspicion of any form of glaucoma or eye disease were placed in the control group. We determined the severity of the glaucoma according to the Hodapp-Anderson-Parrish (HAP) grading system in the study group [16]. Early glaucomatous loss was determined when less than 10 points below the p <1 % level, less than 18 points depressed below the 5 % probability level, no point in the central 5 degrees with a sensitivity of fewer than 15 dB, and MD higher than −6 dB; moderate glaucomatous loss was determined when less than 20 points below the p <1 % level, less than 37 points depressed below the 5 % probability level, the MD is between −6 to −12 dB, no absolute defect (0 dB) in the central 5 degrees, only one hemifield with the sensitivity of <15 dB in the central 5 degrees, and the advanced glaucomatous loss was determined when more than 20 points below the *p* < 1 % level, more than 37 points depressed below the 5 % probability level, an MD higher than −12 dB, absolute deficit (0 dB) in the central 5 degrees, and sensitivity less than 15 dB in the central 5 degrees in both hemifields.

Patients compliance to their medication regimen was assessed. Compliance was considered as following the regimen on a daily basis over the past 2 or 3 months. High treatment compliance was defined as missing installation not more than once a week. Poor treatment compliance was defined as missing at least two drop of medication per week and or the inability to accurately describe one’s own medication regimen.

All participants were given the Turkish version of the TCI for evaluating personality [17, 18]. The TCI is a self-reported evaluate with 240 true/false items measuring four domains of temperament; Harm avoidance (HA) includes anticipatory worry (HA1), fear of uncertainty (HA2), shyness (HA3) and fatigability (HA4). Reward Dependence (RD) includes sentimentality (RD1), openness to friendly communication (RD2), attachment (RD3), and dependence (RD4). Novelty Seeking (NS) is contain exploratory excitability (NS1), impulsiveness (NS2), extravagance (NS3) and disorderliness (NS4). Persistence (PS) is also included in the temperament dimensions.

The three domains of character are: self-directedness (SD), cooperativeness (C), and self-transcendence (ST). SD includes responsibility (SD1), purposeful (SD2), resourcefulness (SD3), self-acceptance (SD4), and congruence (SD5). C consists of social acceptance (C1), empathy (C2), helpfulness (C3), compassion (C4), pure-hearted conscience (C5), and ST includes self-forgetful (ST1), transpersonal identification (ST2), and spiritual acceptance (ST3).

The suitability for a normal distribution of quantitative data was analyzed by using the Kolmogorov-Smirnov test. The test was used for the intergroup comparison of variables, which are suitable for normal distribution, and the descriptive statistics were shown as the mean ± standard deviation form. The Intergroup comparison of the variables that were not suitable for a normal distribution was carried out with the Mann Witney U test, and the descriptive statistics were shown as the median (25–75 percentiles) form. The Chi-square test was used for the comparison of the qualitative data. The Spearman correlation analysis was used for the relationship between variables. When the *p* value was < 0.05, it was considered as statistically significant.

## Results

In the glaucoma group, there were 54 male patients and 50 female patients, while the control group consisted of 67 male patients and 63 female patients. The mean age was 59.36 ± 10.38 years and 57.34 ± 8.27 years in the glaucoma and control groups respectively. There was no difference between the demographic variables of the two groups (Table 1).

**Table 1:** Sociodemographic characteristics of glaucoma patients and control subjects

	Glaucoma (*n* = 104)	Control (*n* = 130)	*p*
Age (Mean ± SD)	59.36 ± 10.38	57.34 ± 8.27	0.124
Gender			0.347
Male	50 (48.1 %)	67 (51.5 %)	
Female	54 (51.9 %)	63 (48.5 %)	
Education			0.337
Primary education	58 (55.8 %)	68 (52.3 %)	
Secondary education	29 (27.9 %)	32 (24.6 %)	
High school	17 (16.3 %)	30 (23.1 %)	
Live			0.176
Village or Town	20 (19.2 %)	17 (13.1 %)	
City	84 (80.8 %)	113 (86.9 %)	
Living situation			0.676
Living alone	14 (12.3 %)	18 (13.8 %)	
Living with a partner	100 (87.7 %)	112 (86.2 %)	

The duration of the glaucoma was 61.17 ± 46.47 months in the study group. Seventy nine (76 %) patients had high treatment compliance, and 25 (24 %) patients had poor treatment compliance. Twenty six glaucoma patients (25 %) had undergone previous glaucoma surgery. The details of the ophthalmic examination results in the glaucoma patients are shown in Table 2.

**Table 2:** Details of ophthalmic examination results in glaucoma patients

BCVA OD	0.81 ± 0.27
BCVA OS	0.79 ± 0.30
Duration of illness (month)	61.17 ± 46.47
Glaucoma severity	
• Early glaucomatous loss	40 (38.5 %)
• Moderate glaucomatous loss	48 (46.1 %)
• Advance glaucomatous loss	16 (15.4 %)
Previous glaucoma surgery	26 (25 %)
Treatment compliance	
• High	79 (76 %)
• Poor	25 (24 %)
Antiglaucoma medication	
• One box	34 (32.7 %)
• Two boxes	30 (28.8 %)
• Three boxes	31 (29.8 %)
• No medication	9 (8.7 %)

The mean scores regarding the TCI dimensions among the glaucoma patients and the control subjects are shown in Table 3.

**Table 3:** Mean scores on TCI dimensions among glaucoma patients and control subjects

	Glaucoma (*n* = 104)	Control (*n* = 130)	*P*
Exploratory excitability (NS1)	5 (4–6)	6 (4–7)	0,200
Impulsiveness (NS2)	4 (3–5)	4 (3–5)	0,554
Extravagance (NS3)	3 (2–4)	5 (3–6)	<0,001
Disorderliness (NS4)	3 (2–5)	4 (3–5)	0,006
Total Novelty seeking (NS) score	16 (14–19)	18 (16–21)	<0,001
Anticipatory worry (HA1)	6 (4–7)	5 (4–6)	0,048
Fear of uncertainty (HA2)	5 (4–6)	4 (3–5)	<0,001
Shyness (HA3)	4 (3–6)	3 (2–4)	<0,001
Fatigability (HA4)	4 (3–6)	4 (3–6)	0,259
Total Harm avoidance (HA) score	19 (17–22)	17 (14–19)	<0,001
Sentimentality (RD1)	7 (5–8)	7 (6–8)	0,280
Openness to warm communication (RD2)	4 (3–6)	4 (3–6)	0.259
Attachment (RD3)	4 (3–5)	4 (3–5)	0,762
Dependence (RD4)	2 (2–4)	3 (2–4)	0,166
Total Reward dependence (RD) score	13 (12–15)	13 (11–15)	0,944
Persistence	5 ( 3, 25–5, 75)	6 (4–7)	<0,001
Responsibility (SD1)	5 (3–6)	4 (3–5)	0,392
Purposeful (SD2)	5 (4–6)	5 (4–7)	0,795
Resourcefulness (SD3)	3 (2, 25–4)	3 (2–4)	0,617
Self-acceptance (SD4)	6 (5–7)	5 (4–7)	0,116
Congruence (SD5)	8 (7–10)	8 (6–9)	0,008
Total Self-directedness (SD) score	27 (24–30)	25 (23–29)	0,033
Social acceptance (C1)	5 (4–6)	6 (5–6)	0,026
Empathy (C2)	4 (2–4)	4 (3–5)	0,014
Helpfulness (C3)	4 (3–5)	4 (3,25-5)	0,925
Compassion (C4)	8 (6–9)	7 (6–9)	0,413
Pure-hearted conscience (C5)	6 (5–7)	6 (5–7)	0,168
Total Cooperativeness (C) score	26 (23–28)	26, 5 (24–30)	0,343
Self-forgetful (ST1)	6 (4–7)	6 (5–7)	0,286
Transpersonal identification (ST2)	5 (4–7)	6 (5–7)	0,054
Spiritual acceptance (ST3)	6 (5–7)	7 (5, 25–9)	<0,001
Total Self-transcendence (ST) score	17 (15–19)	19 (16–22)	0,002

The glaucoma patients had significantly lower scores than the controls on NS and also on the two NS dimensions: NS3 and NS4. The patients with glaucoma had higher scores than the controls HA (*p* < 0.001) and the three HA dimensions; HA1, HA2 and HA3 (*p* = 0.048, *p* < 0.001 and *p* < 0.001). There was no difference according to the RD and C scores between the groups (*p* = 0.944 and *p* = 0.343). PS was significantly lower in the glaucoma patients than in the control subjects (*p* < 0.001). The glaucoma patients had significantly higher scores than the controls in SD (*p* = 0.033) and one SD dimension: SD5 (*p* = 0.008). The patients with glaucoma had significantly lower scores than the controls in ST (*p* = 0.02) and one ST dimension: ST3 (*p* < 0.001). There was no statistical difference in the TCI according to the gender in the groups.

The correlations between the TCI dimensions and the duration of the illness, the treatment response, the disease severity, the adherence to glaucoma medication, and previous glaucoma surgery among the glaucoma patients are shown in Table 4.

**Table 4:** Associations between TCI and duration of illness, disease severity, previous glaucoma surgery, treatment response in glaucoma patients

		Duration of illness	Disease severity	Previous glaucoma surgery	Treatment response
NS1	r	0,071	−0,197	0.096	−0,003
	p	0,472	0,045	0.334	0,979
NS2	r	0,113	−0,029	0.094	0,046
	p	0,254	0,773	0.341	0,643
NS3	r	0,036	−0,028	−0.084	0,075
	p	0,716	0,780	0.395	0,451
NS4	r	0,041	0,056	0.055	−0,019
	p	0,679	0,576	0.577	0,850
NS Total	r	0,160	−0,105	0.079	0,058
	p	0,106	0,288	0.425	0,560
HA1	r	−0,086	0,227	−0.089	0,190
	p	0,383	0,021	0.368	0,054
HA2	r	−0,128	0,056	−0.220	0,147
	p	0,194	0,576	0.025	0,137
HA3	r	0,078	0,057	-.163	−0,028
	p	0,432	0,567	0.097	0,778
HA4	r	−0,047	0,083	0.140	−0,037
	p	0,633	0,403	0.156	0,712
HA Total	r	−0,080	0,220	−0.084	0,097
	p	0,419	0,025	0.395	0,328
RD1	r	−0,189	0,012	−0.254	0,127
	p	0,055	0,906	0.009	0,199
RD2	r	−0,165	0,056	−0.254	0,055
	p	−0.143	0,854	0.234	0,188
RD3	r	0,062	−0,025	−0.133	0,036
	p	0,531	0,802	0.177	0,720
RD4	r	0,043	0,017	0.099	0,067
	p	0,668	0,860	0.319	0,498
RD Total	r	−0,084	−0,021	−0.244	0,145
	p	0,394	0,835	0.013	0,141
P	r	−0,027	0,076	−0.222	0,187
	p	0,785	0,441	0.023	0,057
SD1	r	−0,046	0,005	0.072	−0,052
	p	0,646	0,960	0.470	0,597
SD2	r	−0,156	−0,155	−0.042	−0,008
	p	0,115	0,117	0.670	0,937
SD3	r	0,088	−0,045	0.247	−0,182
	p	0,376	0,651	0.011	0,064
SD4	r	−0,081	0,052	−0.91	0,056
	p	0,411	0,603	0.359	0,571
SD5	r	−0,042	−0,009	−0.076	0,021
	p	0,673	0,926	0.444	0,829
SD Total	r	−0,070	−0,037	−0.005	−0,029
	p	0,481	0,706	0.958	0,771
C1	r	0,028	−0,074	−0.014	−0,005
	p	0,781	0,458	0.892	0,961
C2	r	0,055	0,029	0.301	−0,094
	p	0,581	0,770	0.002	0,342
C3	r	−0,036	0,042	−0.056	0,087
	p	0,719	0,674	0.570	0,378
C4	r	−0,103	0,018	−0.203	0,226
	p	0,298	0,858	0.039	0,021
C5	r	−0,084	−0,071	−0.201	−0,009
	p	0,395	0,476	0.041	0,926
C Total	r	−0,107	0,015	−0.100	0,134
	p	0,281	0,877	0.312	0,175
ST1	r	0,005	−0,021	0.016	0,168
	p	0,962	0,834	0.871	0,088
ST2	r	−0,158	0,015	−0.141	0,258
	p	0,109	0,880	0.154	0,008
ST3	r	0,025	0,028	0.017	−0,035
	p	0,800	0,774	0.865	0,723
ST v Total	r	−0,043	0,021	−0.072	0,172
	p	0,662	0,834	0.469	0,081

Exploratory excitability (NS1), Impulsiveness (NS2), Extravagance (NS3), Disorderliness (NS4), Novelty seeking (NS), Anticipatory worry (HA1), Fear of uncertainty (HA2), Shyness (HA3), Fatigability (HA4), Harm avoidance (HA), Sentimentality (RD1), Attachment (RD3), Dependence (RD4), Reward dependence (RD), Persistence (P), Responsibility (SD1), Purposeful (SD2), Resourcefulness (SD3), Self-acceptance (SD4), Congruence (SD5), Self-directedness (SD), Social acceptance (C1), Empathy (C2), Helpfulness (C3), Compassion (C4), Pure-hearted conscience (C5), Cooperativeness (C), Self-forgetful (ST1), Transpersonal identification (ST2), Spiritual acceptance (ST3), Self-transcendence (ST)

There was no correlation between the duration of the illness and the TCI dimensions. The disease severity was negatively associated with NS1 (*r* = −0,197, *p* = 0,045) and positively associated with HA1 (*r* = 0.227, *p* = 0.021) and HA (*r* = 0,220, *p* = 0,025). The treatment response was associated with the C4 (*r* = 0,226, *p* = 0,021) and ST2 (*r* = 0,258, *p* = 0,008) dimensions. There were associations between the previous glaucoma surgery and seven TCI dimensions also; HA2 (*r* = −0.220, *p* = 0.025), RD1 (*r* = −0.254, *p* = 0.009), RD (*r* = −0.244, *p* = 0.013), P (*r* = −0.222, *p* = 0.023), SD3 (*r* = 0.247, *p* = 0.011), C2 (*r* = 0.301, *p* = 0.002) and C4 (*r* = −0.203, *p* = 0.039).

## Discussion

This study showed that the glaucoma patients have a characteristic personality profile with their lower scores for the NS, P, ST, and higher scores for the HA and SD dimensions when compared to the controls. Temperament is described as a heritable individual difference, and in particular, HA is viewed as a heritable bias in anxiety, which is evidenced by anticipatory worry, shyness, and increased fatigability, all in response to signals of punishment. The disease severity was positively associated with the HA and HA1 dimension as well as the higher scores in the HA in the glaucoma patients than in the controls. Neuroticism is expressed as subscales of the Neuroticism Extraversion Openness Five-Factor Inventory (NEO-FFI) developed by Eysenck, and designed to measure how the subject can cope with, and beat stress as in HA [19]. High HA scores were found to show a strong correlation with high Neuroticism scores [20]. High neuroticism scores are the most consistent findings in glaucoma patients in studies investigating personality in glaucoma [4, 21, 22].Recently, Bubella et al. [13] investigated the type A personality type, which is a more stress-sensitive trait, and they found that type A behavior is much more evident in glaucoma patients. They demonstrated that patients with type A behavior have more fluctuations in the daily tonometric curve, which could be the cause of the more evident field defects [13]. Our findings suggested that higher HA personality features may be associated with glaucoma in accordance with the publications such as higher neuroticism and type A behavior, and also may create a predisposition for glaucoma. These personality features are associated with emotional instability that have been associated with dysregulated intraocular pressure in several studies [13, 23]. Trying to improve the emotional instability may lead to important implications for the long-term therapeutic approach to glaucoma.

As another temperamental dimension, NS was lower in the glaucoma patients than in the controls in our study. NS is characterised by exploration, curiosity, impulsivity and disorganization, and is seen as a tendency to respond to novel stimuli or potential rewards, and actively avoid monotony and punishment [14]. NS is reported as being significantly positively correlated in the personality trait of the extraversion of the Eysenck Personality Questionnaire [19, 24]. Lower extraversion is reported repetitively in different studies in glaucoma patients [4, 21]. Differences found in the temperament dimensions NS and HA in our glaucoma patients may reflect their impulsive behavior and automatic decision-making behavior decrease, whereas fearfulness and social inhibition become more pronounced than in the controls.

PS was significantly lower in the glaucoma patients than the control subjects in our study. Persistence is defined as a tendency to persevere in behaviors implicated with reward or relief for punishments [14]. A negative correlation between the harm avoidance and persistence dimensions were demonstrated with a metaanalysis conducted by Miettunen et al. [25] Increased HA scores in glaucoma patients may affect the persistence dimension in our study according to the literature [25].

Character is referred to as a self-concept, and is affected by life experiences and susceptible to learning. Thus, becoming more flexible and thereby configuring individual differences in goals and values. However, the temperament dimensions of personality are defined as being of genetic and biologic structure [17]. Character is defined by three components; SD, C and ST. [17] C was compared between the patients and controls, and there was no difference. However, the glaucoma patients had significantly higher scores than the controls in SD. The higher SD scores reported that increased coherence of personality or ‘maturity’, protected individuals from depression [26]. This finding was also replicated by Cloninger et al. [26] and to cope with having a chronic illness this component may be elevated in patients. ST is defined as a character trait associated with spirituality by Cloninger et al. [14, 17] When comparing ST, the patients with glaucoma had significantly lower scores than the controls. The lower ST in glaucoma patients may lead them away from spirituality; making them more realistic than the rest of the population.

There was no relationship between the disease duration and temperament with the character dimensions in the current study. Personality is generally accepted as a stable structure, and according to Cloninger’s model is thought to have a strong long-term stability. Our findings may not support the hypothesis that chronic diseases may lead to personality change in different ways, according to Cloninger’s model. As supported by other studies in the literature, characteristic personality profiles in patients can facilitate the occurrence of disease in susceptible individuals [10, 13].

There was no difference in the personality dimension between men and women according to some studies [4]. Mabuchi et al. [5] investigated personality in glaucoma patients by using the NEO-FFI, and they revealed the characteristic personality profile (higher neuroticism, lower extraversion, agreeableness, and conscientiousness) occurred in men rather than women. Bubella et al. [13] reported that there was type A behavior in both sexes, but to a higher level in women.

There were some differences in the personality of patients who had previously had glaucoma surgery than with those who had not. HA2, RD1, RD, P and C4 were lower, SD3 and C2 were higher in the patients with glaucoma surgery than those without. These differences may be risk factors that predispose to surgery or that may be developed after the surgical procedure for adaptive reasons.

This study should be regarded as an initial exploration of the personality structure of Turkish POAG patients. However, caution should be exercised when interpreting the findings due to limitations and confounding factors. Firstly, the sample size of this study was relatively small. Secondly, psychiatric symptoms, anxiety, and depression levels of the patients were not further questioned by clinicians, and not included in the study. More studies and different psychological testing methods are required for more conclusive results.

According to Cloninger’s model research finding demonstrated that a most common personality profile of chronic disease in aged population patients (different form of chronic pain, ischemic heart disease, chronic obstructive lung disease, hypertension) characterized by prevailing harm avoidance which has been shown to predict the presence of a personality disorder as like our findings [27–31]. This patients could benefit from the measurement of personality by the temperament and character inventory and psychological interventions for improved treatment response.

## Conclusion

In conclusion, our findings indicate that significant differences were found between POAG patients and controls for temperament and character personality features (TCI 240); the glaucoma patients had lower scores for the NS, P, ST, and higher scores for the HA and SD dimensions than the controls. It is suggested that personality factors may be closely related to POAG, and that the personality features of patients should be taken into account when treating those with glaucoma.

## Abbreviations

TCI: Temperament and Character Inventory
HA: Harm avoidance
PS: Persistence
NS: Novelty seeking
RD: Reward dependence
ST: Self-transcendence
C: Cooperativeness
SD: Self-directedness
IOP: Intraocular pressure
POAG: Primary Open-Angle Glaucoma
